# Comparative transcriptome profiling of *Eimeria tenella* in various developmental stages and functional analysis of an ApiAP2 transcription factor exclusively expressed during sporogony

**DOI:** 10.1186/s13071-023-05828-8

**Published:** 2023-07-19

**Authors:** Linlin Chen, Xinming Tang, Pei Sun, Dandan Hu, Yuanyuan Zhang, Chaoyue Wang, Junmin Chen, Jie Liu, Yang Gao, Zhenkai Hao, Ning Zhang, Wenxuan Chen, Fujie Xie, Xun Suo, Xianyong Liu

**Affiliations:** 1 National Key Laboratory of Veterinary Public Health Security, Key Laboratory of Animal Epidemiology and Zoonosis of Ministry of Agriculture, National Animal Protozoa Laboratory & College of Veterinary Medicine, China Agricultural University, Beijing, 100193 China; 2 Key Laboratory of Animal Biosafety Risk Prevention and Control (North) of MARA, Institute of Animal Sciences, Chinese Academy of Agricultural Sciences, Beijing, China; 3 School of Animal Science and Technology, Guangxi University, Nanning, China; 4 Key Laboratory of Animal Genetics, Breeding and Reproduction of the Ministry of Agriculture & Beijing Key Laboratory of Animal Genetic Improvement, China Agricultural University, Beijing, China; 5 Department of Pathogen Biology, Guangdong Provincial Key Laboratory of Tropical Disease Research, School of Public Health, Southern Medical University, Guangzhou, China

**Keywords:** *Eimeria tenella*, Life cycle, Transcriptome, ApiAP2 transcription factor, Gene editing

## Abstract

**Background:**

The apicomplexan parasites *Eimeria* spp. are the causative agents of coccidiosis, a disease with a significant global impact on the poultry industry. The complex life cycle of *Eimeria* spp. involves exogenous (sporogony) and endogenous (schizogony and gametogony) stages. Unfortunately, the genetic regulation of these highly dynamic processes, particularly for genes involved in specific developmental phases, is not well understood.

**Methods:**

In this study, we used RNA sequencing (RNA-Seq) analysis to identify expressed genes and differentially expressed genes (DEGs) at seven time points representing different developmental stages of *Eimeria tenella*. We then performed K-means clustering along with co-expression analysis to identify functionally enriched gene clusters. Additionally, we predicted apicomplexan AP2 transcription factors in *E. tenella* using bioinformatics methods. Finally, we generated overexpression and knockout strains of ETH2_0411800 to observe its impact on *E. tenella* development.

**Results:**

In total, we identified 7329 genes that are expressed during various developmental stages, with 3342 genes exhibiting differential expression during development. Using K-means clustering along with co-expression analysis, we identified clusters functionally enriched for oocyte meiosis, cell cycle, and signaling pathway. Among the 53 predicted ApiAP2 transcription factors, ETH2_0411800 was found to be exclusively expressed during sporogony. The ETH2_0411800 overexpression and knockout strains did not exhibit significant differences in oocyst size or output compared to the parental strain, while the resulting ETH2_0411800 knockout parasite showed a relatively small oocyst output.

**Conclusions:**

The findings of our research suggest that ETH2_0411800 is not essential for the growth and development of *E. tenella*. Our study provides insights into the gene expression dynamics and is a valuable resource for exploring the roles of transcription factor genes in regulating the development of *Eimeria* parasites.

**Graphical Abstract:**

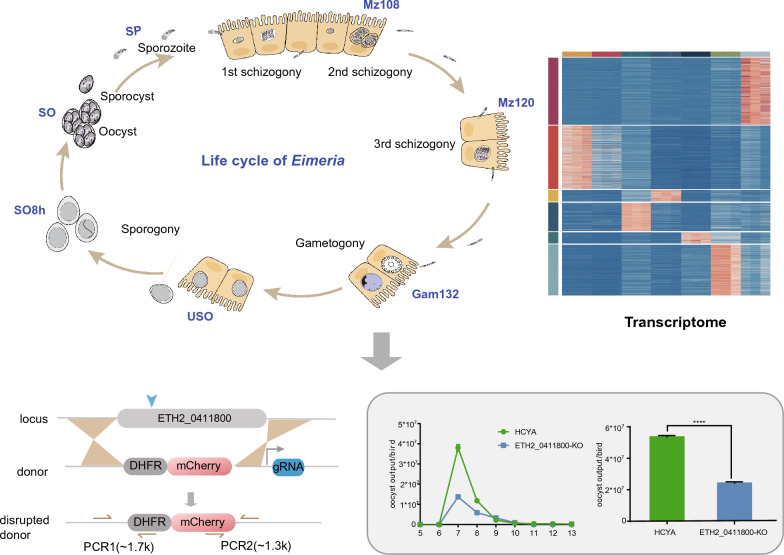

**Supplementary Information:**

The online version contains supplementary material available at 10.1186/s13071-023-05828-8.

## Background

Chicken coccidiosis, caused by the infection of apicomplexan parasites belonging to the genus *Eimeria*, leads to significant economic losses to the poultry industry [1, 2]. The current measures for the control of chicken coccidiosis largely rely on the use of anticoccidial drugs or live vaccines [3–5]. Among the seven *Eimeria* species that infect chickens, *Eimeria tenella* is the most pathogenic and prevalent species [1, 6]. *Eimeria tenella* is also a model species for investigating the biology of *Eimeria* parasites.

The life cycle of eimerian parasites comprises three developmental stages: the exogenous sporogony of oocysts and the endogenous schizogony and gametogony [7, 8]. Although previously reported studies have aptly described the appearance and characteristics of parasites sampled from these developmental stages through electron microscopy [9–11], little is known about the regulatory mechanisms governing the switching of different developmental stages. Since the proliferation and differentiation of parasites during these stages are strictly governed by specific sets of genes, comprehending their biology through the perspective of gene regulation is essential. Taking advantage of genome and transcriptome sequencing [12–14], the dawn of deciphering the genetic regulation of development in eimerian parasites appears on the horizon. RNA sequencing (RNA-Seq) technology is widely used to assess expression patterns of genes in different developmental stages [15, 16], as well as in exploring the differentially expressed genes (DEGs) among samples with different phenotypes such as drug-sensitive or drug-resistant [17–19]. To date, hundreds to thousands of stage-specific genes have been identified in *Eimeria* spp., but only a few of them have been demonstrated to be useful markers for particular stages.

In the present study, we obtained gene expression profiles for exogenous sporogony, endogenous schizogony, and gametogony stages of *E. tenella* at seven time points, including unsporulated oocysts (USO), partially sporulated (8 h) oocysts (SO8h), sporulated oocysts (SO), sporozoites (SP), merozoites (Mz108 and Mz120), and gametocytes (Gam132). Among the DEGs, we focused on ApiAP2 transcription factor (TF) genes and investigated the function of ETH2_0411800 through gene editing. Our findings contribute to a much more detailed understanding of the biological processes of *E. tenella*, which is crucial for developing new interventions and treatments to control coccidiosis in poultry.

## Methods

### Animals and parasites

One- to 6-week-old Arbor Acres (AA) broilers were purchased from Beijing Arbor Acres Poultry Breeding Co., Ltd. (Beijing, China), and 1-week-old specific-pathogen-free (SPF) chickens were purchased from Beijing Boehringer Ingelheim Vital Biotechnology Co., Ltd. (Beijing, China). All chickens were raised in isolators equipped with high-efficiency particulate air (HEPA) filters and were fed coccidia-free feed and water. The *E. tenella* Houghton (ETH) strain used in this study was kindly provided by Professor Damer Blake of the Royal Veterinary College, University of London. A Cas9-expressing transgenic *E. tenella* strain (HCYA) was used for gene editing [20].

### Preparation of samples for RNA-Seq at seven time points

Unsporulated oocysts (USO) were collected from the cecal contents of 20 2-week-old AA broilers at 7 days post-infection (dpi) and then purified [21]. The purified oocysts were subjected to sporulation at 28 °C in 2.5% potassium dichromate. Partially sporulated oocysts (SO8h) were collected 8 h after the sporulation process, and completely sporulated oocysts (SO) were collected after 48 h [22]. Sporozoites (SP) were harvested from sporulated oocysts after in vitro excystation with bile–trypsin treatment and Percoll gradient centrifugation [21].

For the collection of merozoite or gametocyte samples, eight 6-week-old AA broilers were infected with 10^5^ sporulated oocysts of ETH. Merozoites were isolated at 108 h (Mz108) and 120 h (Mz120) from the ceca of chickens following previous studies [16, 23]. In brief, the contents scraped from the cecal mucosa were subjected to treatment using 0.5% sodium taurodeoxycholate hydrate and 0.25% trypsin in phosphate-buffered saline (PBS) for 30 min at 42 °C. The suspension containing free merozoites was filtered through gauze and then centrifuged at 3600 rpm for 5 min. Then the parasite pellets were washed and suspended with PBS. Cecal mucosa containing gametocytes of three SPF chickens (Gam132) were collected at 132 h post-infection [24].

### RNA-Seq, read mapping, and data processing

Total RNA was extracted with Trizol™ reagent (Thermo Fisher Scientific) from the above samples. RNA concentration and quality were then assessed using a NanoDrop ND-2000 and the Agilent RNA 6000 Nano Kit on a 2100 Bioanalyzer instrument (Agilent, USA). The different sequencing libraries were constructed using the TruSeq RNA Library Prep Kit (Illumina, USA) and sequenced on an Illumina NovaSeq platform, and 150-base-pair (bp) paired-end reads were generated. RNA-Seq was conducted by Novogene Co, Ltd. (Beijing, China).

Low-quality reads and adapter sequences were trimmed using Trimmomatic-0.38. The clean reads were mapped to the chromosome-level *E. tenella* reference genome (ToxoDB-60_EtenellaHoughton2021, https://toxodb.org/toxo/app/downloads/Current_Release/EtenellaHoughton2021/) using STAR 2.5.3a [25] and read counting was performed using featureCounts, and then transcript abundance in transcripts per million (TPM) was calculated from featureCounts output [26]. Principal component analysis (PCA) was performed using the PCAtools [27] to exhibit the correlation of transcriptomes. Hierarchical clustering analysis (HCA) was performed with pheatmap [28]. Clustering of transcripts was performed using the K-means clustering algorithm with manual adjustment.

Differential expression of the transcripts was analyzed using the likelihood ratio test (LRT) in the DESeq2. The resulting lists of DEGs were sorted and filtered with a false discovery rate (FDR) threshold of less than 0.01. Gene Ontology (GO) and Kyoto Encyclopedia of Genes and Genomes (KEGG) enrichment analysis was conducted using the R package “clusterProfiler.” The threshold significance of the *P*-value uses FDR calibration at 0.05.

### Genome-wide prediction of ApiAP2 TFs

To identify ApiAP2 TFs genes in *E. tenella* genomes, we collected all protein sequences annotated in the *E. tenella* genomes to form an initial set of protein sequences. The TFs were gathered from information obtained from the PlantTFDB 5.0 plant transcription factor database, Human TFDB 3.0, and AnimalTFDB 3.0. In parallel, a custom hidden Markov model (HMM) profile for TF DNA-binding domains (DBDs) was generated and used to scan the proteomes of *E. tenella* using hmmsearch (HMMER V.3.3) with an E-value cutoff of 1e-5. The National Center for Biotechnology Information (NCBI) Conserved Domain Search service (CD Search) was used to manually confirm the predicted TFs.

### Construction of ETH2_0411800 knockout and overexpression parasites

The ETH2_0411800 knockout parasites were constructed by clustered regularly interspaced short palindromic repeats (CRISPR)/CRISPR-associated protein 9 (Cas9)-mediated gene editing of the ETH2_0411800 locus in the HCYA strain of *E. tenella*. In the knockout plasmid, mCherry was fused to the pyrimethamine-resistant gene TgDHFR-ts-m2m3 (DHFR), which was flanked by homologous sequences of 800 bp derived from 5′ and 3′ of ETH2_0411800. The guide RNA (gRNA) target to the AP2 domain of ETH2_0411800 was fused into the single-guide RNA (sgRNA)-expressing cassette driven by EtU6. For overexpressing ETH2_0411800 in the Houghton strain of *E. tenella*, the EYFP gene fused to DHFR was ligated to the 3′ of ETH2_0411800 CDS, which was flanked by a Flag-tag at each end. The transfection, selection by pyrimethamine and sorting with fluorescence-activated cell sorting (FACS), and the propagation of the transgenic parasites were performed following our previous protocol [20].

### Biological characterization of ETH2_0411800 overexpression and knockout parasites

To investigate the impact of ETH2_0411800 on the life cycle of *E. tenella*, the oocyst output was compared between ETH2_0411800 overexpression and the wild-type parent strain, as well as between ETH2_0411800 knockout and HCYA strains. One-week-old AA broilers (*n* = 3) were infected with 1000 fresh oocysts. Feces were collected daily from infected birds during the period 5–12 dpi. The daily oocyst output was measured with a McMaster chamber for the detection of oocyst shedding. After sporulation, the oocyst size (both the length and width) was measured under a microscope. Cecal samples of one-week-old AA broiler chickens (*n* = 3) were collected at 108 h, 120 h, and 136 h after infection with ETH2_0411800 knockout strain and subjected to preparation of sections for H&E staining.

### Immunofluorescence assay

Human foreskin fibroblast (HFF) cells infected with sporozoites of ETH2_0411800 overexpression strain for 12 h and grown on coverslips were fixed in 4% paraformaldehyde for 1 h at 37 °C, followed by permeabilization with 0.25% Triton X-100 (Sigma, MC0711) for 20 min and blocking with 3% bovine serum albumin (BSA). Slides of parasites-infected cells were then incubated with anti-Flag antibody (1;1000, Sigma, RRID [research resource identifier]: AB_1960908), anti-Cy3 antibody (1:200, Proteintech, RRID: AB_10892835), and Hoechst (1;200, Macgene, RRID: AB_2651133) for 1 h. After washing three times in PBS, the slides were sealed with antifade mounting media. Images were captured with a fluorescence microscope (IX71, Olympus).

### Western blotting

Immunoblot analysis of proteins was performed with ~ 10^7^ sporozoites of ETH2_0411800 overexpression strain or ETH in lysis buffer, respectively. Proteins were separated using sodium dodecyl sulfate–polyacrylamide gel electrophoresis (SDS-PAGE) and transferred to a polyvinylidene fluoride (PVDF) membrane. The blocked PVDF membranes were probed with anti-Flag antibodies (1:1000, Sigma, RRID: AB_1960908), anti-GAPDH (glyceraldehyde-3-phosphate dehydrogenase) antibodies (1:50000, Proteintech, RRID: AB_2107436), and anti-mouse secondary antibodies (1:2000, Macgene, RRID: AB_895481). Signals were detected using a Tanon 1600 (Tanon, China).

## Results

### Characteristics of transcriptomes of development stages in *E. tenella*

To investigate the dynamics of gene expression by transcriptomic analysis, we sampled a total of seven time points covering the three developmental stages of *E. tenella*. At each time point, three biological replicates of parasite samples were collected, of which 30–90% of reads were mapped to the *E. tenella* reference genome (Additional file 1: Table S1). We only used unique mapped reads to calculate normalized gene expression in terms of TPM. To study the relationship between samples and evaluate the reproducibility of the biological replicates, we performed a Pearson correlation analysis matrix of the samples and found that the biological replicates showed good reproducibility. Transcriptomes collected from close time points were observed to have a high degree of similarity (Additional file 8: Figure S1). The transcripts with a TPM equal to or greater than one TPM in at least two of the three biological replicates were considered as expression.

**Fig. 1:**
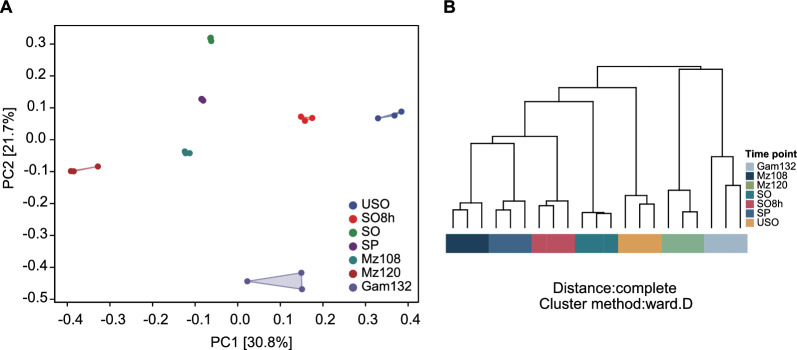
Transcriptome relationships of *E. tenella* at different developmental time points. **A** PCA of the transcriptomes of *E. tenella* developmental stages: unsporulated oocysts (USO), partially sporulated oocysts (SO8h), sporulated oocysts (SO), merozoites (Mz108 and Mz120), and gametocytes (Gam132). **B** Hierarchical clustering analysis of seven time points. Genes expressed in at least one stage (TPM ≥ 1) were used for PCA and hierarchical clustering by utilizing a Z-score normalization step

To gain insight into the transcriptome dynamics during *E. tenella* development, we performed PCA and HCA. The PCA plot showed the top two principal components that explain most of the variance between samples in the data set, 30.8% and 21.7% for PC1 and PC2, suggesting that the major source of the variances among the samples is due to samples from different time points. Seven time points were clustered away from each other (Fig. 1A). Based on HCA, the expression profiles of the seven time points are separated (Fig. 1B). Mz120 was more highly correlated with Gam132 than Mz108.

### Functional enrichment of expressed genes identified during seven time points of* E. tenella*

**Fig. 2:**
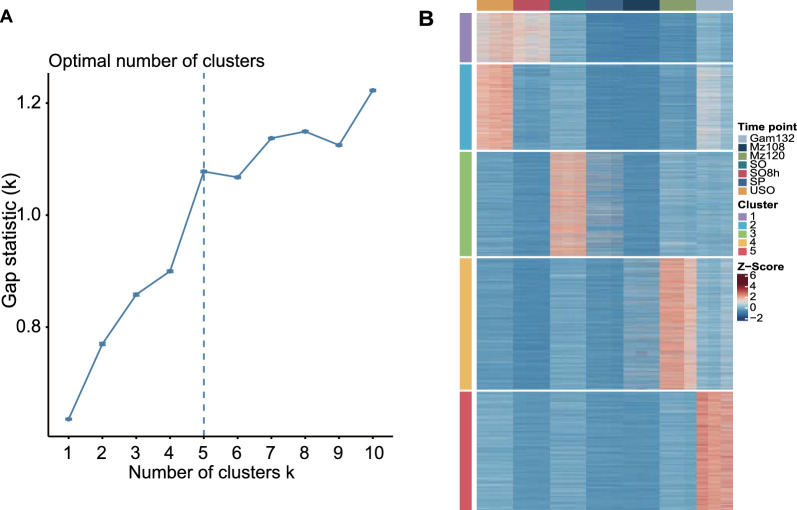
Gene expression patterns during *E. tenella* development. **A** Determining the optimal number of clusters for K-means clustering. The factoextra package embedded in R was used to determine the optimal number of clusters. The dashed line indicates the optimal number of clusters. **B** The expression profile of transcripts during development. The expression of transcripts during development was grouped into 5 clusters. At each stage, the data were shown in three biological replicates

**Fig. 3:**
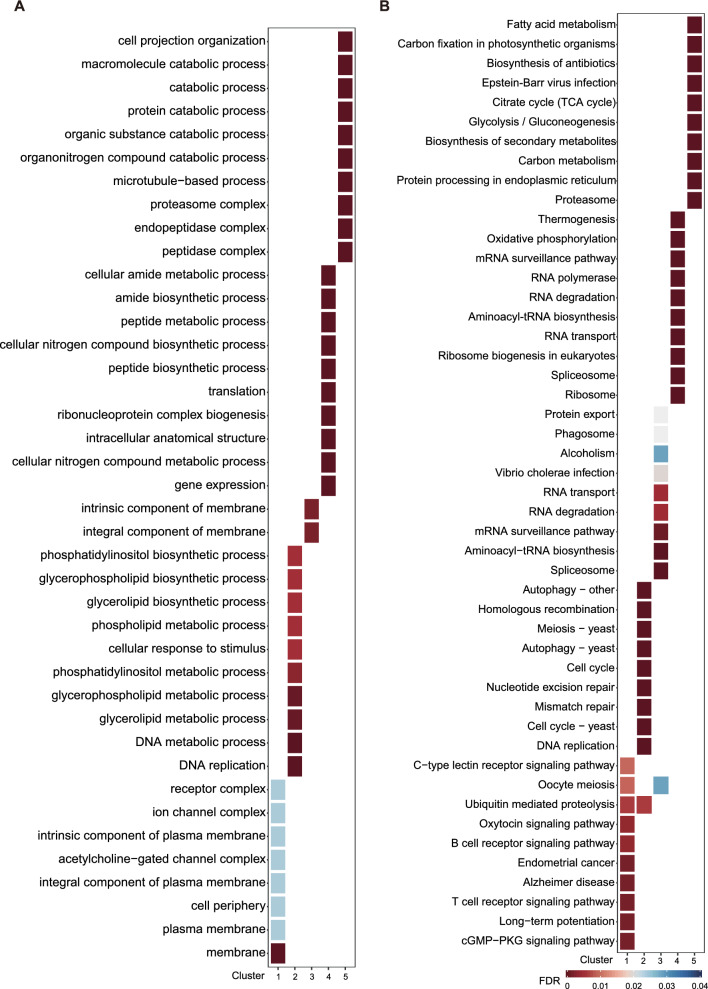
Functional enrichment analysis in five co-expression clusters during *E. tenella* development. **A** GO enrichment analysis and **B** KEGG enrichment analysis based on the development process. ClusterProfiler was used for functional enrichment analysis, with all genes as background. A hypergeometric test was carried out, and all significant categories (false discovery rate < 0.05) are displayed

A total of 7329 expressed genes (Additional file 2: Table S2) of all the samples were clustered into five groups using co-expression patterns detection (Fig. 2A and B, Additional file 3: Table S3). Cluster 2 was found to be highly expressed during the stage of unsporulated oocysts, represented by 1281 genes (Fig. 2B; Additional file 3: Table S3). The genes in cluster 2 were mainly enriched in GO terms “DNA replication,” “metabolic processes,” and “biosynthetic processes” (Fig. 3A). A total of 55 KEGG pathways were identified, among which the top 10 pathways were found to be significantly enriched in processes such as DNA replication, cell cycle, DNA repair, and meiosis (Fig. 3B), highlighting the complex regulatory mechanisms involved in the development of unsporulated oocysts.

The development process of sporogony was represented by 737 genes highly expressed at USO and SO8h time points in cluster 1 (Fig. 2B; Additional file 3: Table S3). Genes highly expressed at this process were enriched in GO terms “membrane” (Fig. 3A; Additional file 4: Table S4). Genes highly expressed during sporogony were primarily assigned to the KEGG pathways of “signaling pathway” (cyclic guanosine monophosphate-protein kinase G [cGMP-PKG] signaling pathway, calcium signaling pathway, and cyclic adenosine monophosphate [cAMP] signaling pathway) and “oocyte meiosis” (Fig. 3B; Additional file 4: Table S4), indicating a complex regulatory network in the sporogony process.

Cluster 3, comprising 1562 genes, displayed high expression levels during the sporulated oocyst stage (Fig. 2B; Additional file 3: Table S3). The highly expressed genes in this cluster were found to be mainly associated with the GO terms “integral component of membrane” and “intrinsic component of membrane” (Fig. 3A; Additional file 4: Table S4). Genes were primarily linked to KEGG pathways like “spliceosome,” “RNA biosynthesis,” and “oocyte meiosis” (Fig. 3B; Additional file 4: Table S4).

Cluster 4, consisting of 1968 genes, was the cluster that best represented the process of schizogony (Fig. 2B; Additional file 3: Table S3). Enrichment analysis revealed that this cluster was particularly significant for “gene expression,” “compounds metabolic and biosynthetic processes,” and “translation.” On the other hand, cluster 5, composed of 1781 genes, was predominantly expressed at Gam132 (Fig. 2B; Additional file 3: Table S3). The highly expressed genes in this cluster were mainly related to GO terms such as “peptidase complex,” “microtubule-based process,” and “catabolic process” (Fig. 3A; Additional file 4: Table S4).

### Global comparison of transcriptomic change during *E. tenella* developmental stages

To identify DEGs during development, we used the DESeq2 for differential expression analysis under stringent criteria of FDR < 0.01. We identified the 3342 DEGs in response to developmental processes (Additional file 5: Table S5), reflecting the great difference across the different developmental stages. We performed a K-means clustering analysis of all DEGs to identify co-expression patterns that may inform future functional studies. We determined the optimal number of clusters to be six clusters that emerged from the cluster analysis (Additional files 5 and 9: Table S5 and Figure S2).

Cluster 1 includes genes that are upregulated at the gametocyte stage and generally downregulated throughout the other stages. The biological process microtubule-based process was highly enriched in cluster 1. Cluster 2 includes genes that are upregulated during the process of sporogony but are then consistently downregulated during endogenous parasite development. For cluster 2, enrichment analysis indicated significant enrichment of the DNA metabolic process (Additional file 10: Figure S3B). KEGG pathway analysis showed that the DEGs were significantly enriched in DNA replication, oocyte meiosis, and cell cycle (Additional file 6: Table S6). Clusters 3 and 4 include genes that are separately upregulated at the sporozoite and sporulated oocysts stage. The cluster group (clusters 5 and 6) includes genes that are upregulated during schizogony. Cluster 4 includes genes that are upregulated at the sporulated oocyst stage. Enrichment analysis indicated significant enrichment of the “integral component of membrane” and “intrinsic component of membrane” (Additional file 10: Figure S3C). Clusters 5 and 6 include genes, expressed in schizogony, which showed enrichment of core functions such as “ribosome,” “RNA binding,” and “gene expression” (Additional file 10: Figure S3D).

### Expression dynamic of ApiAP2 TFs

**Fig. 4:**
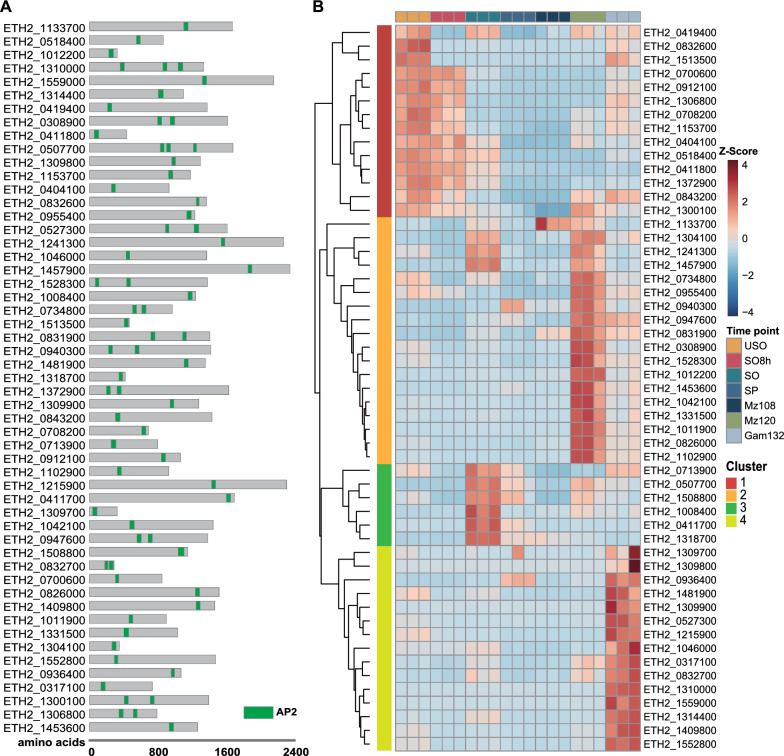
Prediction and validation of expression patterns of 53 ApiAP2 transcription factors in *E. tenella*. **A** Gene IDs of the 53 ApiAP2 transcription factors in the *E. tenella* genome, their respective protein architecture schematic (with AP2 domain displayed in green). **B** Heatmap representation of expression patterns for ApiAP2 genes at seven time points. The Z-score was calculated for the normalized expression of ApiAP2 TF genes

ApiAP2 TFs play a crucial role in regulating gene expression in apicomplexan parasites, specifically during stage-specific transitions that occur throughout their life cycle. Previous studies have highlighted the essentiality of these TFs in regulating development. We identified 53 AP2 domains-contained proteins in the *E. tenella* genome (Fig. 4A, Additional file 7: Table S7). To further explore the expression patterns of these TF genes, we analyzed their expression profiles in different development stages of *E. tenella* (Fig. 4B; Additional file 7: Table S7), which demonstrated that particular expression pattern. Heatmap analysis clustered these genes into four major districted clades according to differential expression patterns (Fig. 4B). We observed ApiAP2 genes in cluster 1 highly expressed in oocysts, indicating that these genes played a role in sporogony. Cluster 2 genes are highly expressed in schizogony, and these genes could be further divided into two different clusters, one of which was specifically expressed in Mz120. Genes of cluster 3 displayed higher expression levels in sporulated oocysts. Cluster 4 was best represented by 15 ApiAP2 genes, which are highly expressed at the gametocyte stage. However, the functions of members of the ApiAP2 family remain unknown in *E. tenella*.

### Overexpression and CRISPR/Cas9-mediated knockout of an ApiAP2 gene

**Fig. 5:**
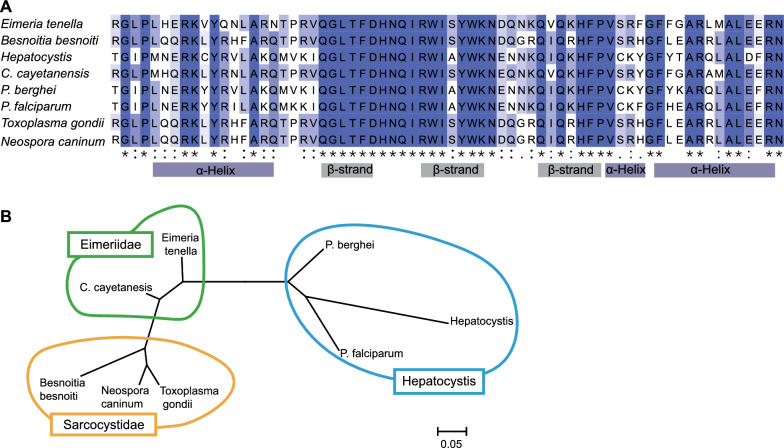
Identification of possible ETH2_0411800 orthologs in apicomplexan parasites. **A** Alignment of conserved amino acid sequences from ETH2_0411800 and BlastP-searched proteins by the ClustalW program in MEGA 11. Positions at which all sequences have an identical amino acid are indicated by one asterisk. Amino acid sequences were retrieved from the PlasmoDB and ToxoDB databases. (*E. tenella*, ETH2_0411800; *Besnoitia besnoiti*, BESB_028780; *Hepatocystis*, HEP_00213800; *C. cayetanensis*, cyc_04832; *P. berghei*, PBANKA_1001800; *P. falciparum*, PF3D7_0404100; *T. gondii*, TGME49_251740; *Neospora caninum*, NCLIV_066800). **B** Phylogenetic tree of ETH2_0411800 and homologous proteins, which were inferred from their whole amino acid sequences using the maximum likelihood method and Jones–Taylor–Thornton (JTT) matrix-based model

ETH2_0411800, which encodes a protein containing one AP2 domain (Fig. 4A), is highly USO-specific (approximately 20-fold higher in USO than Mz108, Mz120, and Gam132) (Additional file 2: Table S2). And this gene was highly expressed during sporogony (Fig. 4B). To investigate whether ETH2_0411800 is conserved in apicomplexan protozoan parasite, we searched for any proteins with a domain similar to the AP2 domain of ETH2_0411800. Protein–protein BLAST (BlastP) search identified proteins with a homologous domain to the AP2 domain of ETH2_0411800 from apicomplexan parasites *Plasmodium falciparum*, *Plasmodium berghei*, and *Toxoplasma gondii* (Fig. 5A). The phylogenetic tree of ETH2_0411800 and the homologous proteins was topologically consistent with that of the tree of Apicomplexa, which further suggested that these proteins are orthologs (Fig. 5B). Collectively, we concluded that ETH2_0411800 is an ApiAP2 family protein conserved in the phylum Apicomplexa.

**Fig. 6:**
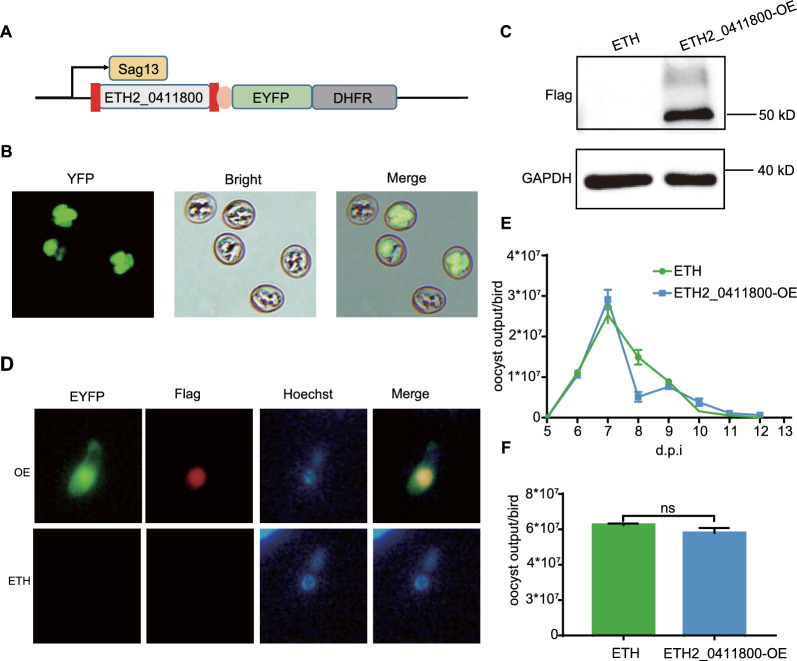
Construction, and phenotyping of ETH2_0411800 overexpression parasites. **A** Schematic representation of ETH2_0411800 gene-overexpressing vector. Two Flag peptides (in red) were fused at the N and C terminal. **B** The expression of ETH2_0411800 (fused upstream of EYFP) in oocysts was determined by fluorescent microscopy. **C** Western blotting showing ETH2_0411800 expression. **D** IFA analysis of the ETH2_0411800 protein in overexpression parasites by using an anti-Flag antibody. The nuclei were visualized by Hoechst staining. **E** Oocyst output curves and **F** total oocyst output of overexpression strain. Chicken (*n* = 3) were infected with 1000 oocysts of the overexpression strain. ETH was used as a control. Error bars represent SEM. *P*-values were obtained using unpaired two-tailed Student’s *t*-tests. *P < 0.05; **P < 0.01; ***P < 0.001; *ns* not significant

To explore how ETH2_0411800 contributes to *E. tenella* development, we generated the ETH2_0411800 overexpression strain (Fig. 6A and B). Western blotting analysis showed the successful expression of ETH2_0411800 in *E. tenella* (Fig. 6C). To further analyze the localization of ETH2_0411800, indirect immunofluorescence assays (IFAs) performed on the stage of SP revealed that ETH2_0411800 localized within the nuclei by co-staining with Hoechst (Fig. 6D). The overexpression strain produced oocysts that were indistinguishable from wide type in size and numbers (Fig. 6B and E). Analysis of the ETH2_0411800-overexpression strain showed a similar oocyst output curve when compared to those of the wild type (Fig. 6E and F).

**Fig. 7:**
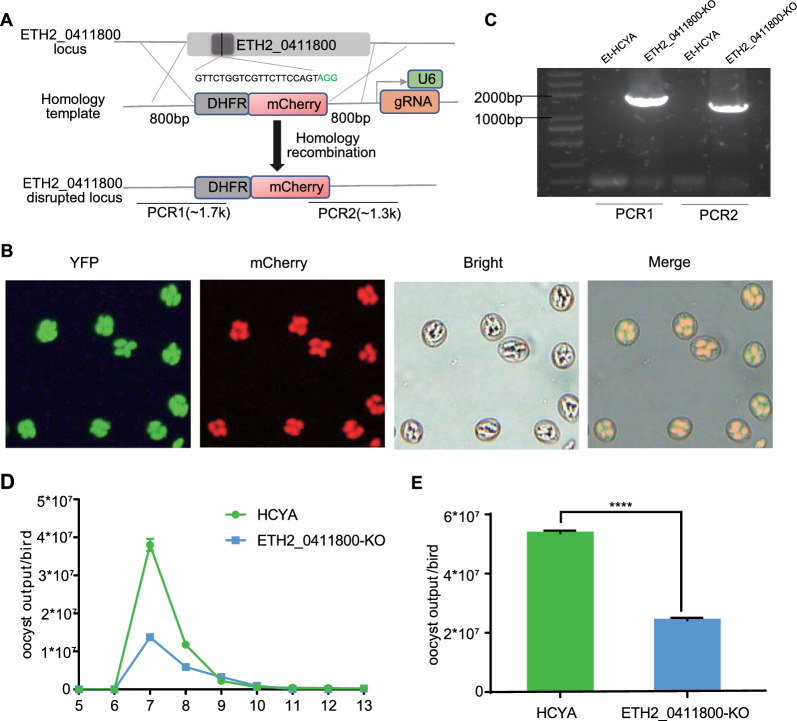
Construction and phenotyping of ETH2_0411800 knockout parasites. **A** Schematic representation of the transfection vector used for disrupting the ETH2_0411800 locus. Homology regions from 5′ (5′HR) and 3′ (3′HR) of the ETH2_0411800 locus were ligated to the expression cassette containing the mCherry fluorescent protein fused to DHFR. **B** The expression of mCherry was determined by fluorescent microscopy. **C** Representative PCR products from a knockout clone for ETH2_0411800. The oligonucleotides were designed from regions located outside 5′HR and 3′HR and within the ETH2_0411800 locus. **D** Oocyst output curves and **E** total oocyst output of knockout parasites. Chicken (*n* = 3) were infected with 1000 oocysts. HCYA was used as a control. The standard error of the mean (SEM) is presented as an error bar. Unpaired two-tailed Student’s *t*-tests were conducted, and the significance level was indicated as ****P < 0.0001

To explore the function of the ETH2_0411800 gene, we used genome editing for the generation of an ETH2_0411800 knockout strain (Fig. 7A and B). The deletion of ETH2_0411800 was validated by polymerase chain reaction (PCR) and sequencing (Fig. 7C). ETH2_0411800 gene knockout parasites showed a comparative growth, i.e., similar oocyst output curve and relatively small oocyst output (Fig. 7D and E). Comparison of endogenous development showed no significant difference compared to the control strain (Additional file 11: Figure S4). These findings indicate that ETH2_0411800 is not crucial for the growth and development of *E. tenella*.

## Discussion

Our study presents a comprehensive transcriptional analysis of gene expression patterns during the developmental stages of *E. tenella*. We identified 7329 genes that are expressed during various developmental stages, with 3342 genes exhibiting differential expression during development, which were thus useful for inferring gene function and understanding the genetic control of developmental transitions. Furthermore, from 53 predicted ApiAP2 TFs, we validated their transcriptional across three stages of the life cycle. We then constructed overexpression and knockout parasites for a sporogony stage-specific ApiAP2 gene, ETH2_0411800, and studied its role in endogenous development.

During endogenous schizogony and gametogony development, parasites switch from asexual replication to sexual development, and the DEGs between these stages are enriched in GO in terms of participating in translation, gene expression, substance biosynthesis, and metabolism processes, as well as microtubule-associated proteins. These results provide insights into the complex biological processes underlying the development of *E. tenella*. Transcriptome analysis at three time points during sporogony showed significant upregulation of genes related to the membrane components, oocyte meiosis, and signaling pathways, which was consistent with results of other *Eimeria* spp. [24, 29]. Based on our analysis, the cGMP-PKG signaling pathway was identified as the most significant pathway, indicating its significant role in the sporogony of *E. tenella*. The parasite's cGMP-PKG signaling pathway is essential at multiple stages of the parasite life cycle [30–33]. Previous research on *Eimeria* transcriptional changes revealed increased expression of gametocyte-specific genes, including GAM56 and HAP2, along with upregulation of hundreds of other genes [16, 34]. The study found upregulation of most of these genes at Gam132, which were specifically related to gametogony. Collectively, these molecular clues unveil the formerly unknown developmental process of *E. tenella* at the molecular level.

Despite extensive research on ApiAP2 TFs in apicomplexan parasites such as *Plasmodium* spp., *T. gondii*, and *Cryptosporidium* [35–40], their function in *Eimeria* pp. has not been studied to date. We also identified 53 ApiAP2 TFs by using a bioinformatics approach. The ETH2 _0411800 was found mainly expressed during oocyst stages, indicating that it is a sporogony stage-specific gene in *E. tenella*. ETH2_0411800 is an AP2-Sp2 homologous expressed exclusively during sporogony. Previous studies showed that AP2-Sp2 together with three other TFs (AP2-Sp, AP2-Sp3, and SLARP) play important roles in regulating gene expression during sporozoite development in malaria [41–44]. ETH2_0411800 knockout parasites grew similarly to HCYA parasites, indicating that this gene may not play a significant role in development. Whether ETH2_0411800 regulates the expression of other genes remains unclear and requires further study.

Our analysis revealed that another ApiAP2 gene ETH2_0940300 is also highly expressed during schizogony (Additional file 12: Figure S5). Its homologous gene, TgAP2XII-1 of *T. gondii*, is expressed during the tachyzoite stage. Furthermore, TgAP2XII-1 works in concert with TgAP2IX-4 and microrchidia (MORC) to regulate the expression of genes critical to cell cycle progression [45]. The latest study demonstrates AP2XII-1 and AP2XI-2 bind cooperatively to DNA as heterodimers and selectively recruit HDAC3 and MORC to the promoter of merozoite genes, which in turn creates a non-permissive chromatin environment for transcription in tachyzoites [46]. These findings suggest that ETH2_0940300 plays a crucial role in regulating the development of parasites by controlling cell cycle-related genes during cell division and development. In this study, we attempted to knock out this ApiAP2 gene using CRISPR/Cas9 but failed (data not shown), which is in accordance with our previous work showing that ETH2_0940300 (ETH_00031200) is indispensable for the survival of the parasites [20]. In the future, a conditional knockout system is needed for the study of essential genes in eimerian parasites.

## Conclusions

Our study provides a comprehensive understanding of transcriptome characteristics during the various development stages in *E. tenella*, including the expression patterns of the ApiAP2 family. The overexpression and knockout of ETH2_0411800, which is a sporogony stage-specific gene, indicated its potential function related to the amount of oocyst output. The expression patterns and stage-specific genes identified in the present study contribute to a better understanding of crucial stages in the *E. tenella* life cycle, and provide comprehensive transcriptomic data for future studies in this field.

## Supplementary Information

**Additional file 1: Table S1. **Summary of read counts, quality control, and read alignment of RNA-seq. Alignment of reads was performed using the STAR.

**Additional file 2:** **Table S2.** The TPM of expressed genes in different parasite replicates.

**Additional file 3: Table S3.** Expression patterns of genes related to parasite development.

**Additional file 4: Table S4.** GO and KEGG functional categories enriched in five co-expression clusters.

**Additional file 5: Table S5.** Expression patterns of DEGs related to parasite development.

**Additional file 6: Table S6. **GO and KEGG functional categories of DE genes enriched in six co-expression clusters.

**Additional file 7: Table S7.** List of ApiAP2 transcription factors expressed in different parasite samples.

**Additional file 8: Figure S1. **Correlation of expression between replications at each time point for* E. tenella*. There are three biological replicates for each sampling time point.

**Additional file 9: Figure S2. **Global comparison of transcriptomic changes during *E. tenella* development. **A** Determining the optimal number of clusters for K-means clustering. The optimal number of clusters was determined using the factoextra package embedded in R. Dashed line indicates the optimal number of clusters. **B** The expression profile of DEGs during development. The expression of transcripts during development was grouped into six clusters.

**Additional file 10:** **Figure S3.** Functional enrichment analysis in six co-expression clusters of DEGs during *E. tenella* development. **A**–**C** GO enrichment analysis based on clusters 1, 2, and 3. **D** GO enrichment analysis based on clusters 5 and 6. ClusterProfiler was used for functional enrichment analysis, with all genes as background. A hypergeometric test was carried out, and all significant categoriesare displayed.

**Additional file 11: Figure S4.** Tissue sections for observing endogenous development in ETH2_0411800 knockout and HCYA strains. The endogenous development of ETH2_0411800 knockout and HCYA strains was observed using H&E stained cecal sections. The black arrow shows mature second-generation schizonts and the red arrow shows gametophytes. The scale bar represents 50 μm

**Additional file 12:** **Figure S5.** Identification of ETH2_0940300 orthologs in apicomplexan parasites. **A** Schematic representation of ETH2_0940300 and homologous genes. The green box shows the AP2 domain. **B** Alignment of conserved amino acid sequences from ETH2_0940300 and BlastP-searched proteins by the ClustalW program in MEGA 11. Positions at which all sequences have an identical amino acid are indicated by asterisks. Amino acid sequences were retrieved from the PlasmoDB and ToxoDB databases.

## Data Availability

The RNA-Seq data for these isolates are available in the NCBI Sequence Read Archive (SRA) database, under accession number PRJNA951257. This study did not generate any custom code. Any additional information required to reanalyze the data reported in this article is available from the lead contact on request.

## Abbreviations

DEGs: Differentially expressed genes
USO: Unsporulated oocyst
SO8h: Partially sporulated (8 h) oocysts
SO: Sporulated oocyst
SP: Sporozoite
Mz108: Merozoites 108 h
Mz120: Merozoites 120 h
Gam132: Gametocytes 132 h
TF: Transcription factor
HEPA: High-efficiency particulate air
dpi: Days post-infection
TPM: Transcripts per million
PCA: Principal component analysis
HCA: Hierarchical clustering analysis
LRT: Likelihood ratio test
DBDs: DNA-binding domains
FDR: False discovery rate
DHFR: Pyrimethamine-resistant gene TgDHFR-ts-m2m3
HFF: Human foreskin fibroblast
IFAs: Indirect immunofluorescence assays
